# Spatial Variation of Arsenic in Soil, Irrigation Water, and Plant Parts: A Microlevel Study

**DOI:** 10.1155/2016/2186069

**Published:** 2016-09-26

**Authors:** M. S. Kabir, M. A. Salam, D. N. R. Paul, M. I. Hossain, N. M. F. Rahman, Abdullah Aziz, M. A. Latif

**Affiliations:** ^1^Bangladesh Rice Research Institute (BRRI), Gazipur, Bangladesh; ^2^Department of Statistics, Jahangirnagar University, Savar, Dhaka, Bangladesh; ^3^Department of Business Administration, Uttara University, Uttara, Bangladesh; ^4^Agricultural Statistics Division, BRRI, Gazipur, Bangladesh; ^5^Plant Pathology Division, BRRI, Gazipur, Bangladesh

## Abstract

Arsenic pollution became a great problem in the recent past in different countries including Bangladesh. The microlevel studies were conducted to see the spatial variation of arsenic in soils and plant parts contaminated through ground water irrigation. The study was performed in shallow tube well command areas in Sadar Upazila (subdistrict), Faridpur, Bangladesh, where both soil and irrigation water arsenic are high. Semivariogram models were computed to determine the spatial dependency of soil, water, grain, straw, and husk arsenic (As). An arsenic concentration surface was created spatially to describe the distribution of arsenic in soil, water, grain, straw, and husk. Command area map was digitized using Arcview GIS from the “mouza” map. Both arsenic contaminated irrigation water and the soils were responsible for accumulation of arsenic in rice straw, husk, and grain. The accumulation of arsenic was higher in water followed by soil, straw, husk, and grain. Arsenic concentration varied widely within command areas. The extent and propensity of arsenic concentration were higher in areas where high concentration of arsenic existed in groundwater and soils. Spherical model was a relatively better and appropriate model. Kriging method appeared to be more suitable in creating interpolated surface. The average arsenic content in grain was 0.08–0.45 mg/kg while in groundwater arsenic level it ranged from 138.0 to 191.3 ppb.

## 1. Introduction

Groundwater from shallow sedimentary aquifers became the principal source for drinking and irrigation in many countries of the world, particularly in the developing countries. In Bangladesh, groundwater is being pumped out by thousands of shallow tube wells (STWs) and a significant number of these wells (around 35%) have been found to contain As above the acceptable limits, but the concentrations in many instances are high (>0.2 mg L^−1^). Agricultural soils in many areas of the country contain total As exceeding 20 mg kg^−1^. There is also growing evidence of elevated accumulation of As in rice, the staple food grain of Bangladesh, and also in other foodstuffs being grown on irrigated land. Bangladesh is one of the most affected countries in the world by As through massive ground water extraction for both drinking and irrigation and is in the midst of what the World Health Organization [1] calls the “largest mass poisoning of population in history.” Since the 1970s, international aid organizations have dug millions of tube wells in Bangladesh for the population to provide safe, bacteria-free drinking water. Moreover, the groundwater has been the main source of irrigation for crops since the mid-1970s as a consequence of the spread of high yielding varieties of crops during the green revolution. This massive country-wide project was often termed the “groundwater revolution” because it saved millions of lives from waterborne diseases such as diarrhea and cholera from the drinking of surface water and stimulated subsequent rapid agricultural growth due to increase of crop land under irrigation. This ground water revolution has now turned into a death trap for many Bangladeshi. Moreover, large areas of Bangladesh have to rely on arsenic contaminated groundwater for irrigation of staple crops such as rice [2, 3]. Irrigation with arsenic contaminated groundwater is leading to elevated levels of arsenic in paddy soils [4] which may lead to increased concentration of arsenic in rice [5, 6], vegetables [7], and other agricultural products of the arsenic affected areas [8]. Ground water is extensively used in irrigation of rice, the staple food of Bangladesh, with 83% of the total irrigated area under rice cultivation [9] and more than 99% of the people eating huge amounts of rice as their main food @455 gm/person/day. Thus, consumption of rice containing a small amount of As may lead to accumulation of large amount of As in human body in long run. This may be equally true for the animals, particularly those that are fed As contaminated rice straw and used for milk and meat. Research has already indicated variation of As concentration in grain and straw in different rice varieties, which again depends on growing environments. Of the three rice seasons, Boro (dry season rice) is fully grown under irrigated condition and 79.1% of the Boro area is irrigated by ground water [10] and, thus, growing rice in the Boro season has become the major concern in terms of arsenic poisoning in human body. Ross et al. [11] reported that mean ground water arsenic concentrations are below 50 *μ*g/L in 76% of irrigated Boro rice areas and in 7% of areas, mean concentrations of As are greater than 100 *μ*g/L primarily in south central and western central Bangladesh. Arsenic in rice grains is especially dangerous for the Bangladeshi people because of the relatively high consumption of about 450 g per person per day. Arsenic contamination of the irrigation water-soil-crop system, especially in the rice systems, is a matter of great concern. We need to substantially improve our understanding of the As problem and protect millions of our fellow human beings from exposure to As by scientifically intervening in its pathways through the sediment-water-soil-crop-human system. We need to pool the knowledge and experiences of chemists, geochemists, biologists, agricultural scientists, and medical scientists to find solutions to a problem that threatens the lives and livelihoods of hundreds of millions of human beings across the world.

Very little is known about the spatial variability of arsenic loading in irrigated and in nonirrigated soil and its impact on arsenic accumulation in rice grain and straw. Understanding of geostatistical processes is very important to know the spatial variability of As in soils. Geostatistical methods can be powerful tools for characterizing large-scale spatial distributions of soil properties for precision agriculture. Geostatistical method has a number of implications. Firstly, it will help to delineate the spatial variability of soil and water arsenic. Secondly, it will help to know the real arsenic situation in Bangladesh; and thirdly, it has a great significance for formulating a future policy to manage arsenic problem. It also provides an advanced methodology which facilitates spatial interpolation and qualification of spatial temporal variability in soil variables and has become a useful tool for the study of spatial uncertainty and hazard assessment [12]. Kriging is a precise estimator for spatial data analysis as it is unbiased and minimizes total uncertainty [13]. Nazari Zade et al. [14] used geostatistics method to study spatial variability of groundwater quality in Balarood plain. Their results showed that spherical model is the best model for fitting on experimental variogram of EC, Cl−, and SO_4_^2−^ variables. Istok and Cooper [15] used Kriging method to estimate heavy metals concentration in groundwater and concluded that Kriging method is the best estimator for spatial prediction of lead. Rizzo and Mouser [16] used geostatistics for analyzing groundwater quality. They used microbial data as an auxiliary variable in co-Kriging method. Their results showed that co-Kriging method has suitable accuracy to estimate groundwater quality. Kriging method has a high accuracy in estimating of TDS in groundwater as reported by Ahmed [17]. Gaus et al. [18] studied groundwater pollution in Bangladesh. They used disjunctive Kriging method to estimate arsenic concentration and to prepare risk map. Their results showed that 35 million people are exposed in high concentration of arsenic (50 ppm). Finke et al. [19] used simple Kriging method to estimate water surface changes in Netherlands and introduced it as a suitable method for mapping of water surface. Barca and Passarella [20] used disjunctive Kriging and simulation methods to make nitrate risk map in 10, 50 (mgL^−1^) thresholds, in Modena plain of Italy. Their results showed that disjunctive Kriging method is the suitable method to study deterioration level of groundwater. Because of various results, it is obvious that the suitable method of interpolation to estimate a variable depends on variable type and regional factors; thus, any selected method for specific region cannot be generalized to others. The present study was therefore, carried out with objectives to describe the spatial variation of arsenic in rice straw, husk, and grain in Faridpur in Bangladesh and also to identify appropriate semivariogram model for describing spatial dependency of rice straw, husk, and grain arsenic.

## 2. Materials and Methods

### 2.1. Study Approach

**Figure 1:**
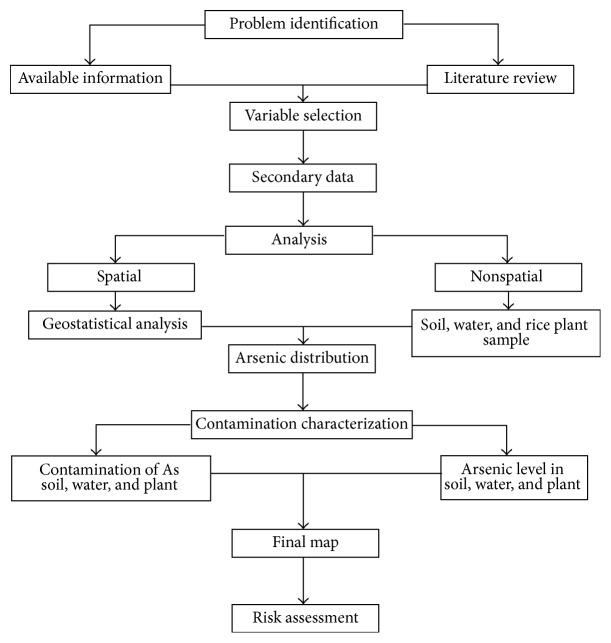
Study approach: Spatial distribution and risk assessment from soil, water, and plant part As.

The study was conducted in a four-phase approach as described in Figure 1.

### 2.2. Data Sources and Sampling Techniques

Impact of arsenic contamination on agricultural sustainability and food quality was determined in Bangladesh jointly by the national partners BRRI, BARI, BAU, and BINA and overseas partners Texas A&M and Cornell University of USA in 2004 under the USAID funded project. Faridpur Sadar Upazila, Bangladesh, was selected purposively where water and soil As was higher among the As affected “upazilas” (subdistricts) in Bangladesh.

### 2.3. Micro Level Data

The shallow tube well (STW) command areas in Kanaipur, Faridpur Sadar Upazila, Faridpur were selected randomly and digitized from the mouza (a mauza is a specific land area within which there may be one or more settlements) maps. Soil samples were collected from the command area at approximately 20 m grids and water samples from the irrigation channel at 20 m interval at random. Rice plant samples were collected randomly from the same grid during harvesting time of 1 m^2^ areas. All samples were georeferenced using Global Positioning System (GPS). Laboratory analysis of the samples was done and values of the soil, ground water, straw, husk, and grain arsenic were recorded.

### 2.4. Description of Study Areas

The study was conducted in a shallow tube well (STW) command area where water and soil As were higher. The site was selected for the study where the level of arsenic in groundwater is frequently exceeding WHO's permissible limit (0.01 mg L^−1^) for drinking water and FAO's permissible limit for irrigation water (0.10 mg L^−1^). The soil As was very high in varying levels in the study areas. The command areas were screen-digitized from mouza maps using Arcview 3.2 and built-in Arcinfo environment. The intensive samples were collected from the four command areas. The description of the four command areas is described below.

**Figure 2:**
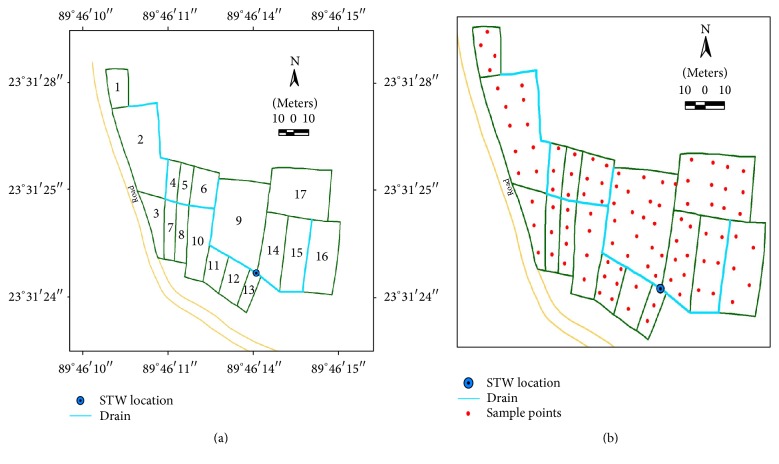
The base map (a) and distribution of the sample points (b) in Kanaipur command area, Faridpur Sadar Upazila.

The command area is Kanaipur, which is 7 km south west from Faridpur Sadar Upazila, Faridpur. Soil and water As was relatively high in this command area. It was located within 23°31′23′′ and 23°31′29′′N latitude and 89°46′10′′ and 89°46′15′′E longitude (Figures 2(a) and 2(b)). The land type of the command area was medium high land and the soil texture was clay loam to loam. The cropping pattern was rice-rice-fallow.

### 2.5. Sample Collection

Irrigation water samples were collected from the source shallow tube well and from the irrigation channel at 20-meter and one-hour interval at random. Prior to sample collection, the pumps were kept running for about 10–15 minutes in order to get a uniform rate of discharging water. Then, the water samples were collected in plastic bottles and preserved with concentrated HNO_3_. At the time of sampling, irrigated Boro rice was in the field. Soil samples were collected from the fields irrigated with the arsenic contaminated water and transferred to airtight polyethylene bags. Composite (three) soil samples were collected from 0–45 cm depth in a 1-x1-m area in 2004 in collaboration with BRRI, BINA, and CIMMYT under USAID funded project. Each sample point was georeferenced using GPS. Rice plant samples were collected from a selected plot of 1 m^2^ areas during harvesting time. The sample size of the command area was 101 (Figure 2(b)). Distributions of the sampling points were covered in the field quite evenly for all command areas. The average sampling interval was 12.35 m over an area of 1.54 ha. The smaller sampling intervals were chosen to resolve any short-scale variation.

**Figure 3:**
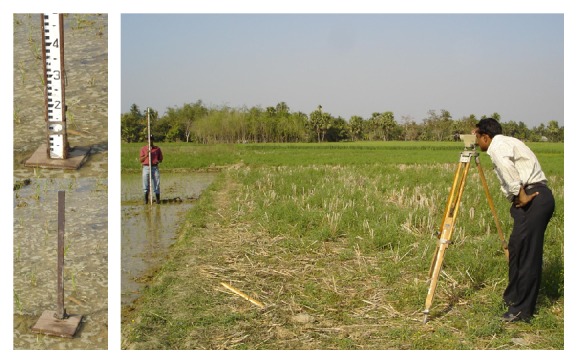
Theodolite used to measure microelevation.

For microelevation model, the elevation at 315 points was measured within the command area including 101 sampling points which were used for soil As using Theodolite (Figure 3). A wooden platform with 6 × 6-inch platform was used to mount the staff in order to avoid the depression of soil surface which was created due to foot pressing during transplanting and other cultural operations in the field. The microelevation model was defined separately within each plot because the variation in elevation was more between plots than within plot. The soil of unit plots was well plowed and leveled prior to transplanting.

### 2.6. Sample Treatment

The irrigation water samples were filtered using 0.45 millipore filter paper and were kept in polyethylene bottles at 4°C for analysis. After collection, soil samples were sun-dried immediately and were dried in the Hot Air Oven at 60°C for 72 hours. The dried soil samples were grinded and passed through 2.0 mm pore sized sieve to get homogenized representative powder samples. The rice plant samples were washed thoroughly with tap water to remove soil and other contaminants and finally rinsed with deionized water with continuous shaking for several minutes. The samples were then dried in the oven at 60°C for 72 hours and were stored in airtight polyethylene bags at room temperature. Proper care was taken at each step to avoid contamination.

### 2.7. Sample Digestion

Soil as well as root, straw, husk, and grain portions of the rice samples were digested separately following heating block digestion procedure. About 0.5 g of the sample was taken into clean dry digestion tubes and 5 mL of concentrated HNO_3_ was added to it. The mixture was allowed to be kept overnight under fume hood. In the following day, the digestion tubes were placed on a heating block and heated at 60°C for 2 hours. The tubes were then allowed to cool at room temperature. About 2 mL of concentrated HClO_4_ was added to the plant samples. For the soil samples, 3 mL of concentrated H_2_SO_4_ was added in addition to 2 mL of concentrated HClO_4_. After that, the tubes were heated at 160°C for about 4-5 hours. The heating was stopped when the dense white fume of HClO_4_ was emitted. The content was then cooled, diluted to 25 mL with deionized water, filtered through Whatman number 42 filter papers for soil samples and Whatman number 41 filter papers for plant samples, and finally stored in plastic bottles. Prior to digestion of samples, all glassware was washed with 2% HNO_3_ followed by rinsing with deionized water and drying.

### 2.8. Descriptive Statistics of Water, Soil, Straw, Husk, and Grain Arsenic

“Boro” (dry season rice) rice cultivation mostly depends on groundwater in Faridpur as the surface water sources (river, dam, pond, etc.) become dry throughout the season. But groundwater is highly contaminated with arsenic. Thus, there is a possibility of induction of arsenic in rice through contaminated ground irrigation water and soil. In this study, samples of irrigation water were collected from the source (shallow tube well) and from the irrigation channel in 20-meter interval. Soil, straw, husk, and grain samples were collected from the field of the command area and analyzed in laboratory.

### 2.9. Selection of the Best Semivariogram Model

There is no standard methodology for choosing among valid variogram models like the spherical or the exponential ones [21]. Least squares technique can be used to fit the selected parametric model, for example, weighted least squares [22] or generalized least squares [23, 24]. The parametric model can then give an estimate of the nugget, sill, and range. Any of the parameters (nugget effect, range, and sill) for each model may be changed within the ranges. The best model for fitting experimental variogram was selected based on the highest *R* ^2^ (regression coefficient), the lowest residual sum of squares (RSS) and the proportion of spatial structure close to unity [22, 24]. The quality of the semivariogram fit to the data was indicated using a regression coefficient, *R* ^2^, and an *F*-test calculated as [25]

(1) $$\begin{array}{c} F=\frac{R^{2}}{1-R^{2}}\times \frac{n-k}{k-1}, \end{array}$$

where *k* is the number of parameters in the regression model and *n* is the number of samples.

## 3. Results and Discussion

### 3.1. Outliers' Analysis

**Figure 4:**
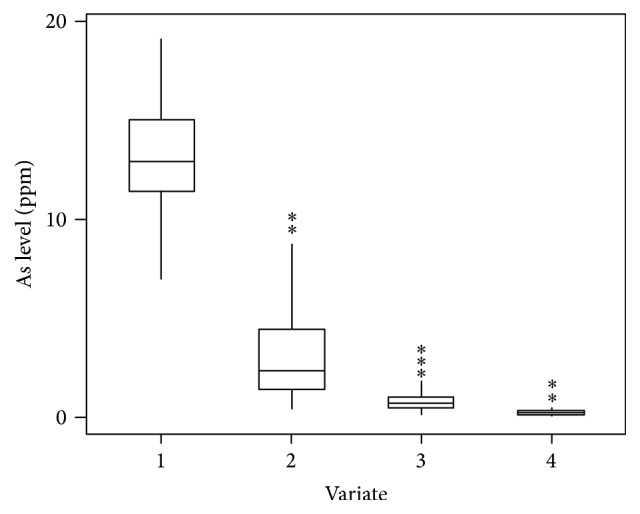
Outliers' in the variate: 1, soil As, 2, straw As, 3, husk As, and 4, grain As which is denoted by (*∗*).

The outlier was removed according to Paul [26]. After removing the identified outliers, all the subsequent analyses were done. Applying the above rule, outliers identified by box plot graph are shown in Figure 4 for soil, straw, husk, and grain As using Minitab 2000 software. No outlier was detected in soil As but two outliers in straw As (9.9 and 10.1 ppm), three in husk As (2.2, 2.5 and 2.7 ppm), and two in grain As (0.53 and 0.99 ppm) were detected.

### 3.2. Descriptive Statistics of Water, Soil, Straw, Husk, and Grain Arsenic

The descriptive statistics of water, soil, straw, husk, and grain As are shown in Table 1.

For agricultural soil, acceptable arsenic limit is 20.0 ppm as recommended by the European Community [27–29]. The World Health Organization [1] safe limit for As in drinking water is 10 *μ*g L^−1^. Food and Agriculture Organization [30] permissible limit for irrigation water is 0.10 ppm [31]. No critical value of straw and husk As has yet been determined. According to WHO recommendation, the permissible limit in rice grain is 1.0 ppm [27–29].

Results revealed that the average As concentration in groundwater was 163.8 ppb ranging from 38.0 to 191.3 ppb which was much higher than that of WHO permissible limit for drinking and irrigation water (Table 1). Similarly, the concentration of As in soil was 13.15 ppm ranging from 6.99 to 18.99 ppm which did not exceed the critical level (>20 ppm).

The straw As was 2.89 ppm which ranged from 0.46 to 9.50 ppm. Similarly, the value for husk was 0.75 ppm which ranged from 0.14 to 1.87 ppm. The mean concentration of arsenic in rice grain was found as 0.23 ppm and ranging from 0.08 to 0.45 ppm. The values did not exceed the permissible limit in rice (1.0 ppm) according to WHO recommendation [27–29]. The accumulation of arsenic in water and soil and in the various parts of rice plant was found in following order: water > soil > straw > husk > grain (Table 1). This as seems an indication that arsenic may transmit through water to soil to straw to husk to grain. However, the results revealed that arsenic supplied by irrigation water was accumulated less in rice grains. The outer fraction of rice (husk) might act as a barrier for the translocation of arsenic. Besides, long term use of arsenic contaminated groundwater for irrigation may result in the further increase of arsenic concentration in the agricultural soil and eventually hyperaccumulation in crops including rice plants.

Relatively good accumulation of arsenic has been observed in its straw and husk portion. Thus, severe health hazard to the large population of cattle and poultry in the study area might occur due to consuming the straw part of the rice plant and husk of grain. The communities of the area are used to eating beef as a readily available and cheap source of meat. The cow milk has been serving the protein requirement of people of all the religions. Poultry meat and eggs are also eaten as a source of protein. Thus, there is a further risk of entering arsenic to human bodies as reported by Meharg and Rahman [5]. Besides, the consumption of rice containing a small amount of As may lead to accumulation of large amount of As in human body in the long run.

Median value of water As also exceeded the critical value (WHO and FAO limit) in the command area. The value of soil As was lower than the critical value (20 ppm); the straw As was 2.25 ppm. The values of husk and grain were 0.65 ppm and 0.22 ppm, respectively (Table 1). The mean was higher than median value which indicated that the influence of a few samples existed with high As levels. Comparing the standard deviations with means, the distributions were not symmetric [32]. Standard deviation of Grain As in Faridpur was close to 1/3 of mean. All other values of standard deviation were not equal to 1/3 of mean indicating the nonnormality of the distribution of As data. According to [33], values of skewness and kurtosis were close to 0 and 3, respectively, indicative of a normal distribution. The skewness and kurtosis of As data are presented in Table 1. The value of skewness of water As was close to 0. Skewness of soil As 0.09 was also close to 0. All other data on As were strongly skewed (>1). In case of kurtosis, none of the coefficient of kurtosis values was close to 3. Nonnormality of the distribution of As data in terms of skewness and kurtosis is an indication of the need for transformation of data. The required transformation was determined using GS+5.3.2 software based on the criteria having skewness close to 0 and kurtosis close to 3 presented in Table 1. From the table, it was revealed that no transformation was needed in water and soil As, LN transformation was needed for straw while SQRT was needed for husk and grain As.

The coefficient of variation (CV) was classified as ≤10% low, 10–20% medium, 20–30% high, and ≥30% very high. CV values are presented in Table 1. Very high variability was observed in soil, straw, husk, and grain, ranging from 34.85 to 69.36% and water and soil were medium. The soil management may have contributed to the great variability of the data in the study area because distribution of irrigation water was not homogeneous due to the lack of plot leveling. This fact caused a modification of the natural spatial variability of soil elements.

### 3.3. Relationship of Water and Soil Arsenic with Distance

**Figure 5:**
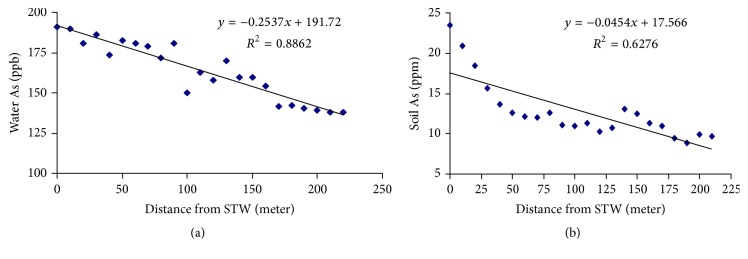
(a) Relationship between water As and distance from the source (STW). (b) Relationship between soil As and distance from the source (STW).

The results of both water and soil samples collected from source are shown in Figures 5(a) and 5(b). The results revealed that the concentration of arsenic in water-flow through the irrigation channel reduced with the distance from the tube well (Figure 5(a)). Similar pattern was observed for As loading in soil; soil As decreased with the increase in distance from the tube well (Figure 5(b)), indicating that a considerable amount of arsenic in water was absorbed by soils while flowing through the channel. The absorption of As was higher near the source (STW) than at the tail end of the irrigation channel. This indicates that As loading in soils from irrigation water at any point in a well leveled command area is spatially related to its distance from the tube well.

### 3.4. Percent Concentration of As in Water, Soil, Straw, Husk, and Grain Samples in the Command Area

Concentration of As in water, soil, straw, husk, and grain samples is presented in Table 2. The hundred percentages of water samples contained As between 100 and 200 ppb. In case of soil samples, 8% contained As less than 10 ppm and 92% contained between 10 and 20 ppm. In Faridpur, 13% straw samples contained As less than 1 ppm, 30% contained between 1 and 2 ppm, and 57% contained more than 2 ppm. In the case of husk, 78% of samples contained As less than 1.0 ppm and 22% contained between 1 and 2 ppm. 40% grain samples contained As less than 0.2 ppm and 60% contained between 0.2 and 0.5 ppm. From the above results, it seems that 100% of the water sample was highly contaminated (>100 ppb) which indicates that most probably water As is responsible for accumulation of As in soil, straw, husk, and grain.

### 3.5. Relationship among Soil, Straw, Husk, and Grain Arsenic 

**Figure 6:**
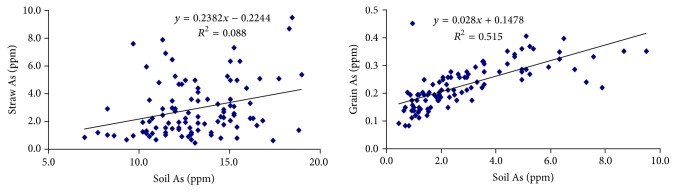
Relationship of soil As with straw and grain As.

In Figure 6, the significant and positive correlation coefficient of soil As was found with straw and grain As. It is evident that higher accumulation of As in straw and grain resulted from the higher concentration of As in soil. Nevertheless, the results indicated differential effect of varieties on the translocation of soil As into straw and grain and this information may be an important input to the researcher for identification and development of As tolerant varieties and in the quest of finding means of mitigating As problem in rice soils.

### 3.6. Relationship between Soil As and Soil Parameters

Relationship of soil As, organic matter (OM), and soil texture is presented in Table 3. It was observed that the soil As content decreased with increase of soil OM. Besides, soil As content was higher for soils with higher silt and lower for soils with higher clay content.

### 3.7. Selection of the Best Semivariogram Model

**Figure 7:**
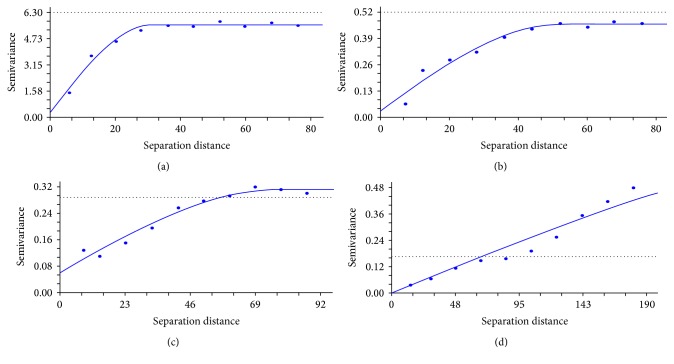
(a) Semi-variogram models for soil As. (b) Semivariogram models for straw As. (c) Semivariogram models for husk As. (d) Semivariogram models for grain As.

After normalizing data using required transformation (Table 2), experimental variograms were computed. Parameters of variograms for soil, straw, husk, and grain in different command areas are presented in Tables 4(a), 4(b), 4(c), and 4(d). Semivariogram model was calculated using GS+ 2000 software. The results showed that, based on the criteria used to judge the adequacy of a model, the spherical model, in general, fitted well to almost all the cases. From some previous studies [34–36], spherical model was found to be the most used in computing semivariograms of soil and plant parts. The semivariograms of soil, straw, husk, and grain As for all the command areas were, therefore, computed using spherical model and are presented in Figures 7(a), 7(b), 7(c), and 7(d).

### 3.8. Parameters of the Semivariogram Models

#### 3.8.1. Range of the Semivariogram Models

Range is separation distance beyond which the variogram value remains essentially constant, that is, at which points in the modeled domain are no longer spatially correlated. The range values (a) of semivariograms for soil, straw, husk, and grain are presented in Tables 4(a), 4(b), 4(c), and 4(d) and Figures 7(a), 7(b), 7(c), and 7(d). Range provided a distance beyond which the variogram value remained essentially constant and within the range it varied from point to point. The ranges of spatial dependency of soil As within the command area were about 31.1 m. For straw, husk, and grain As, the ranges were 52.0, 74.4, and 410.9 m, respectively.

#### 3.8.2. Lag Distance of the Semivariogram Models

The active lag distance specifies the range over which semivariance was calculated. Usually, lag distance is the function of distance *γ*(*h*) which increases with *h*, indicating more deviation and less correlation between *z*-values with increasing distance. The lag distances are presented in Tables 4(a), 4(b), 4(c), and 4(d) and Figures 7(a), 7(b), 7(c), and 7(d). For soil, straw, husk, and grain As the values of lag distance were 80, 80, 92, and 190 m, respectively, indicating As was not so disperse with increasing distance.

#### 3.8.3. Lag Class Distance Interval

The lag class distance interval defined how pairs of points were grouped into lag classes. Each point in a variogram represented the average semivariance for a single lag class, which was a group of pairs separated by a certain lag class distance interval. Sometimes it was called a step size. This interval can either be calculated by GS+, in which case it will be uniformly distributed across the active lag distance, or it can be manually set by the user. The default value was 10% of the active lag. The lag intervals are presented in Tables 4(a)–4(d) and Figures 7(a)–7(d). For soil, straw, husk, and grain As, the lag intervals were 8.0, 8.0, 9.0, and 9.2, respectively. The intervals were uniformly distributed across the active lag distance.

### 3.9. Nugget Effect of the Semivariogram Models

Nugget effect was the nonspatial variability of the variable and was determined when *h* (*h* is the distance between the locations) approaches 0. The nugget effect can be caused by variability at very short distances for which no pairs of observations are available. Sampling inaccuracy or inaccuracy in the instruments was used for measurement. In an ideal case (e.g., where there is no measurement error), the nugget value is zero. Low values of nugget effect (*C*_0_) indicate low errors in measurements [37]. The nugget values are presented in Tables 4(a)–4(d) and Figures 7(a)–7(d). The nugget effect of soil As was 0.29 indicating high analytical error and high variability of soil As within the lag intervals. Similarly, for straw As, the nugget effect was 0.028 which had small analytical error and less variability. It was 0.095 for husk As which was small. For grain As, nugget value was 0.001 indicating small analytical error and less variability. This trend, however, was not consistent in all the plant parts as well as in all variates. It might be due to concentration of the person who analyzed the sample and the variation of reagent concentrations during digestion time.

### 3.10. Sill of the Semi-Variogram Models

The sill of the variogram model (*C*_0_ + *C*) is commonly called the sill. If the variogram reaches sill, semivariance could not be increased with increasing of distance, causing a flat region to occur on the semivariogram. Sill represents spatially independent variance. Data locations were separated by a distance beyond which semivariance did not change. Theoretically, the sill is equivalent to sample variance. The sill values are presented in Tables 4(a)–4(d) and Figures 7(a)–7(d). The sill value of soil, straw, husk, and grain As was 5.55, 0.50, 0.31, and 0.68, respectively, which indicates that, except for grain As, all other variograms reached sill and in these cases semivariance did not increase with distance increases.

### 3.11. Proportion of Spatial Structure of the Semivariogram Models

Proportion of spatial structure or *C*/(*C*_0_ + *C*) provides a measure of the proportion of sample variance (*C*_0_ + *C*) that is explained by spatially structured variance *C*. The proportion of spatial structure to sampling variance was close to unity for As (Tables 4(a)–4(d)) indicating less variability in As within the lag intervals and the semivariogram model explained most of the sampling variation.

### 3.12. Regression Coefficient of the Semivariogram Models

Regression coefficient or *R* ^2^ provides an indication of how well the variogram model fits the data. The values of the regression coefficient are presented in Tables 4(a)–4(d). The values of *R* ^2^ for soil, straw, husk, and grain As were 0.98, 0.96, 0.96, and 0.98, respectively. All the regression coefficients were highly significant at *F*_0.01,*k*−1,*n*−*k*_. It indicated that almost all the cases of spherical model fitted very well for the variogram data, where *k* was the number of parameters in the model.

### 3.13. Residual Sums of Squares of the Semivariogram Models

Residual sums of squares or RSS provide an exact measure of how well the variogram model fits the data; the lower the residual sum of squares, the better the model fit. GS+ uses RSS to choose parameters for each of the variogram models by determining the combination of parameter values that minimizes RSS for any given model. The RSS values are presented in Tables 4(a)–4(d). The minimum value of RSS for soil As was 0.388. Similarly, for straw As RSS was 0.0067 and for husk and grain it was 0.002 and 0.0014, respectively, which indicated almost all the cases of spherical model fitted better for the variogram data.

### 3.14. Akaike Information Criterion or AIC

AIC values provide a means for model selection. AIC not only refers to goodness of fit but also includes a penalty that is an increasing function of the number of estimated parameters. This penalty discourages overfitting. The AIC values are presented in Tables 4(a)–4(d). The lowest value of AIC for soil As was −555.75. Similarly, for straw As, AIC was −965.70 and for husk grain it was −1087.80 and −1123.83, respectively, which indicated spherical model fitted better for variogram data.

### 3.15. Neighborhoods of the Semivariogram Models

The default value of 16 nearest neighbors was usually sufficient, with no restrictions placed on radius. Specifying more than 16 neighbors can slow interpolation substantially. The neighborhoods are presented in Tables 4(a)–4(d). For soil, straw, husk, and grain As, the neighborhoods were 10, 8, 8, and 10, respectively. These determined neighborhoods were considered to estimate the unknown points within known points.

### 3.16. Spatial Dependency of the Semivariogram Models

Spatial dependence is “the propensity for nearby locations to influence each other and to possess similar attributes” [38]. The ratio of nugget variance to sill expressed in percentages can be regarded as a criterion for classifying the spatial dependence of soil, straw, husk, and grain As. If this ratio is less than 25%, then the variable has strong spatial dependence; if the ratio is between 25 and 75%, the variable has moderate spatial dependence and if greater than 75%, the variables show only weak spatial dependence [16, 17]. The ratios of nugget variance to sill are presented in Tables 4(a)–4(d). For soil As ratio of nugget variance was < 25% which exhibited strong spatial dependence among sample points. For straw As, strong spatial dependence was exhibited (6.1%). For husk As, moderate spatial dependence was exhibited (30.6%) and for grain As strong spatial dependence was observed (0.15%). The results indicated that no spatial dependency existed beyond the above ranges of the command area but within command area the nature as well as the extent of dependency was not the same in all variables.

### 3.17. Selection of Interpolation Methods

For determination of the most suitable interpolation method, root mean square error (RMSE) technique was used. The results of RMSE are presented in Tables 5(a) and 5(b). Irrespective of variants RMSE value of Kriging was less than that of IDW except for elevation. Results indicated that geostatistic method (Kriging) was relatively more precise than IDW method. This result was similar to the results of Safari [39], Nazari Zade et al. [14], Ahmed [17], and Barca and Passarella [20].

### 3.18. Spatial Variability of Soil, Straw, Husk, and Grain As

**Figure 8:**
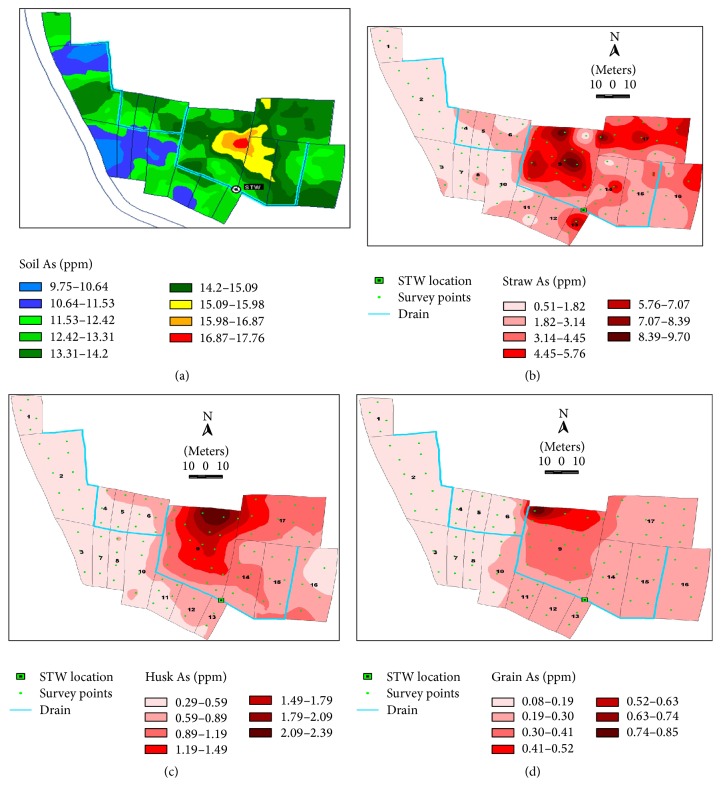
(a) Map of spatial variation of soil As. (b) Map of spatial variation of straw As. (c) Map of spatial variation of husk As. (d) Map of spatial variation of grain As in four command areas.

Interpolated maps of soil, straw, husk, and grain As were prepared using Kriging method. The maps are presented in Figures 8(a)–8(d). In case of soil As, more than 90% of area fell under As level >10 ppm. In straw As, 60% of area had As concentration >2 ppm. Arsenic concentration in husk was more than 2 ppm covered in about 20% of area. About 60% of area showed As concentration in grain >0.2 ppm. The As concentrations varied widely within command area.

Arsenic concentration in soil, grain, and straw was not consistent throughout the command area; rather, As concentration occurred in patches spread over the command area. It was because of As loaded more in the depressed area compared to elevated areas. Besides, due to variability in soil characteristics, a safe level in one soil may be unsafe in a different soil.

### 3.19. Effect of Microelevation on the As Loading in Rice Soils

It was observed during soil sampling that the standing water level in certain areas of some of the rice fields was higher, meaning that the rice fields were not uniformly leveled during land preparation. In other words, there were microdifferences in plot elevation creating some local and patchy depressions within a rice field where irrigation water could stay a long time and the amount of water consumed was higher than that of elevated areas. It was then quite reasonable to hypothesize that As loading in the soil of a rice field from As contaminated irrigation water would be relatively higher at the depressed areas than those of the elevated areas. That microelevation may be an impacting factor for As loading in the groundwater irrigated rice soils.

**Figure 9:**
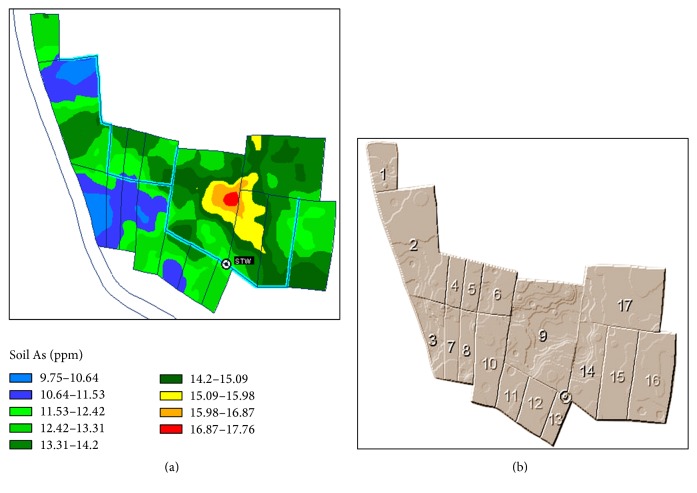
(a) Comparison of soil As surface and elevation model (the number 1,2,… in (b) represents plot number).

In order to test the above hypothesis, microelevation model or microrelief was created for Faridpur command area. The surface was made using IDW interpolation method (Figure 9(b)). The comparison of soil As surface (Figure 9(a)) and elevation model (Figure 9(b)) exhibited close agreement between elevation of the rice field and spatial variability of soil As. In general, soil As was found to be higher in depressed areas than in elevated ones. The correlation coefficient between elevation and soil As within the rice field (Table 6) ranged from −0.58 to −0.82. All were negative and except plot number 11, and all the coefficients were significant at 5% probability level indicating that soil As was expected to be low in high elevated areas.

**Figure 10:**
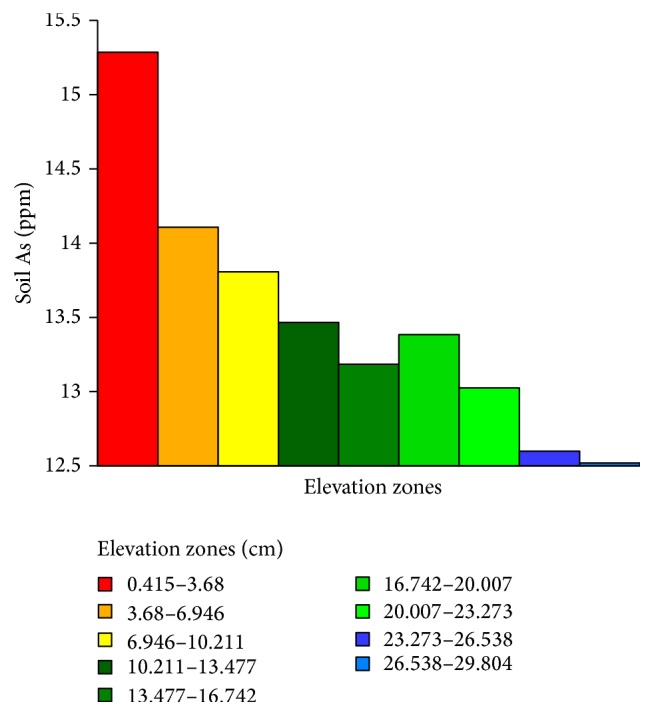
Soil As summarized within the zones of elevation.

Soil As was summarized within the zones of elevation (Figure 10) revealing that As loading in soils was the highest (>15 ppm) in the lowest elevation zone (0.42–3.68 cm) which gradually decreased with the increase in elevation with the only exception in the elevation zone of 16.74–20.01 cm.

## 4. Conclusions

Long-term use of arsenic contaminated groundwater for irrigation may increase the arsenic concentration in the soil and eventually accumulation in edible part of the rice plants. The highest accumulation of arsenic was in water followed by soil, straw, husk, and grain. The rice plant is a good accumulator of arsenic in its straw and husk portion. Arsenic may transmit to food chain through irrigation water and soil and may cause severe health hazard to the cattle and poultry population that creates a further risk of entering arsenic into human bodies. Irrigation water and the soils are responsible for the transfer and uptake of arsenic in rice straw, husk, and grain. The amount, extent, and propensity of As concentration were higher in plant parts, in the areas with high concentration of As in groundwater and soils. The outer fraction of rice (husk) might act as a translocation barrier for not mobilizing As into rice grain. The spherical model, in general, fitted well to almost all the cases in command area. Kriging method appeared to be more suitable in creating interpolated surface for microscale. Microelevation may be an impacting factor for As loading into soil. High organic matter in soil may reduce As accumulation in soils. Long irrigation channels or ponded ground water prior to irrigation and discouraging standing water for long periods in rice fields could be positive measures in reducing arsenic loading in irrigated rice soils. Alternate Wetting and Drying (AWD) method can be a remedial measure to reduce As contamination in soil as well as food chain.

## Figures and Tables

**Table 1:** Descriptive statistics of water, soil, straw, husk, and grain As of Faridpur.

Parameters	Water (ppb)	Soil (ppm)	Straw (ppm)	Husk (ppm)	Grain (ppm)
Mean	163.8	13.15	2.89	0.75	0.23
Median	162.9	12.90	2.25	0.65	0.22
Mode	159.4	13.26	1.19	0.66	0.15
Min	138.0	6.99	0.46	0.14	0.08
Max	191.3	18.99	9.50	1.87	0.45
Sd	1.83	2.51	2.01	0.38	0.08
Skewness	−0.11	0.09	1.18	1.13	0.45
Kurtosis	−1.45	−0.18	0.90	0.87	−0.28
CV (%)	11.16	19.10	69.36	51.14	34.85
Transform	None	None	LN	SQRT	SQRT

**Table 2:** Percent distribution of As concentration in water and soil.

As concentration	
	% of water sample

<50 ppb	0
50–100 ppb	0
100–200 ppb	100
>200 ppb	0

	% of soil sample

<10 ppm	8
10–20 ppm	92
>20 ppm	0

	% of straw sample

<1 ppm	13
1-2 ppm	30
>2 ppm	57

	% of husk sample

<1.0 ppm	78
1.0–2.0 ppm	22
2.0–4.0 ppm	0
>4.0 ppm	0

	% of grain sample

<0.2 ppm	40
0.2–0.5 ppm	60
0.5–1.0 ppm	0
>1.0 ppm	0

**Table 3:** Soil As and textural parameters of surface soil under different command areas.

Sample size (number)	Soil As (ppm)	OM	Soil textural parameters		
			Sand%	Clay%	Silt%
96	9.42	2.10	20.33	26.43	53.24
100	13.15	1.23	24.50	34.42	38.08
60	20.40	1.07	4.98	35.40	56.62
144	8.82	1.99	4.69	85.31	4.00

**(a):** 

Parameters	Spherical	Exponential	Linear	Linear to sill	Gaussian
Range (m)	31.1	39.6	76.1	144.7	25.8
Lag distance	80	80	80	80	80
Lag interval	8	8	8	8	8
Nugget (*C*_0_)	0.290	0.010	3.125	3.140	1.060
Sill (*C*_0_ + *C*)	5.548	5.732	6.379	9.287	5.541
Proportion of structural variance to total sampling variance: *Q* = *C*/(*C* + *C*_0_)	0.95	1.00	0.51	0.66	0.81
*C*_0_/(*C* + *C*_0_)*∗*100	5.0	0.0	49.0	34.0	19.0
*R* ^2^	0.98	0.97	0.58	0.58	0.98
RSS	0.388	0.531	36.7	6.74	0.388
AIC	−555.75	−524.06	−96.25	−267.41	−555.75
Neighborhood	10	10	10	10	10

**(b):** 

Parameters	Spherical	Exponential	Linear	Linear to sill	Gaussian
Range (m)	52.5	75.3	75.8	159.7	44.3
Lag distance	80	80	80	80	80
Lag interval	8	8	8	8	8
Nugget (*C*_0_)	0.028	0.001	0.156	0.158	0.096
Sill (*C*_0_ + *C*)	0.459	0.505	0.535	0.949	0.458
Proportion of structural variance to total sampling variance: *Q* = *C*/(*C* + *C*_0_)	0.94	1.00	0.71	0.83	0.79
*C*_0_/(*C* + *C*_0_)*∗*100	6.0	0.0	29.0	14.0	21.0
*R* ^2^	0.96	0.96	0.79	0.79	0.94
RSS	0.0068	0.0067	0.554	0.0324	0.0088
AIC	−964.20	−965.70	−519.78	−806.52	−938.16
Neighborhood	8	8	8	8	8

**(c):** 

Parameters	Spherical	Exponential	Linear	Linear to sill	Gaussian
Range (m)	74.4	162.3	84.2	183.6	68.2
Lag distance	92.0	92.0	92.0	92.0	92.0
Lag interval	9.2	9.2	9.2	9.2	9.2
Nugget (*C*_0_)	0.059	0.055	0.105	0.107	0.098
Sill (*C*_0_ + *C*)	0.312	0.397	0.349	0.614	0.317
Proportion of structural variance to total sampling variance: *Q* = *C*/(*C* + *C*_0_)	0.81	0.86	0.70	0.83	0.69
*C*_0_/(*C* + *C*_0_)*∗*100	18.9	13.9	30.1	14.4	30.9
*R* ^2^	0.96	0.94	0.88	0.88	0.96
RSS	0.002	0.004	0.225	0.007	0.002
AIC	−1087.80	−1017.79	−610.78	−961.27	−1087.80
Neighborhood	8	8	8	8	8

**(d):** 

Parameters	Spherical	Exponential	Linear	Linear to sill	Gaussian
Range (m)	410.9	1232.7	379.8	379.8	646.4
Lag distance	190	190	190	190	190
Lag interval	19	19	19	19	19
Nugget (*C*_0_)	0.001	0.001	0.001	0.001	0.058
Sill (*C*_0_ + *C*)	0.683	1.154	0.909	0.909	2.126
Proportion of structural variance to total sampling variance: *Q* = *C*/(*C* + *C*_0_)	1.00	0.99	0.99	0.99	0.97
*C*_0_/(*C* + *C*_0_)*∗*100	0	0.1	0.1	0.1	0.3
*R* ^2^	0.98	0.93	0.96	0.96	0.98
RSS	0.0014	0.018	0.011	0.011	0.003
AIC	−1123.83	−865.88	−915.62	−915.62	−1046.85
Neighborhood	10	10	10	10	10

**(a):** 

Soil		Straw	
Kriging	IDW	Kriging	IDW
6.6529	6.6872	0.2636	0.2818

**(b):** 

Husk		Elevation		Grain	
Kriging	IDW	Kriging	IDW	Kriging	IDW
0.4326	0.4333	0.8765	0.8742	0.2356	0.2372

**Table 6:** Correlation between soil As and elevation of the plots.

Plot number	Number of observations	*r*
2	10	−0.79^*∗∗*^
4, 5	6	−0.81^*∗*^
6	6	−0.73^*∗*^
9	20	−0.70^*∗*^
11	6	−0.58^ns^
14	8	−0.79^*∗∗*^
15	6	−0.82^*∗*^
17	12	−0.60^*∗*^

Plots having less than 6 sampling points were excluded from the analysis.

Plots 4 and 5 had similar elevation as indicated by microelevation model.

*∗* and *∗∗* represent significance at 5% and 1% levels. ns means nonsignificant.
